# Numerical Assessment of the Hydrodynamic Behavior of a Volute Centrifugal Pump Handling Emulsion

**DOI:** 10.3390/e24020221

**Published:** 2022-01-31

**Authors:** Lila Achour, Mathieu Specklin, Idir Belaidi, Smaine Kouidri

**Affiliations:** 1 LIFSE, Arts et Métiers Institute of Technology, CNAM, HESAM University, F-75013 Paris, France or l.achour@univ-boumerdes.dz (L.A.); smaine.kouidri@ensam.eu (S.K.); 2 LEMI, FT, University of M’hamed Bougara, Avenue de L’indépendance, Boumerdes 35000, Algeria; i.belaidi@univ-boumerdes.dz

**Keywords:** centrifugal pump, oil–water emulsions, CFD, non-Newtonian, shear-thinning

## Abstract

Although emulsion pumping is a subject of growing interest, a detailed analysis of the fluid dynamic phenomena occurring inside these machines is still lacking. Several computational investigations have been conducted to study centrifugal pumps carrying emulsion by analyzing their overall performance, but no studies involved the rheological behavior of such fluids. The purpose of this study is to perform a computational analysis of the performance and flow characteristics of a centrifugal pump with volute handling emulsions and oil–water mixtures at different water cuts modeled as a shear-thinning non-Newtonian fluid. The studied pump consists of a five-bladed backward curved impeller and a volute and has a specific speed of 32 (metric units). The rheological properties of the mixtures studied were measured experimentally under a shear rate ranging from 1 s^−1^ to 3000 s^−1^ and were fitted to conventional Cross and Carreau effective viscosity models. Numerical results showed the flow topology in the pump is directly related to the viscosity plateau of the pseudoplastic behavior of emulsions. The viscosity plateau governs pump performance by influencing the loss mechanisms that occur within the pump. The larger the ν_∞_, the less recirculation loss the fluid experiences, and conversely, the smaller the value of ν_0_, the less friction loss the fluid experiences.

## 1. Introduction

Centrifugal pumps find application in many engineering processes, chemical processes, wastewater treatment, power generation, and petroleum industries. They are generally designed to transport water or an incompressible fluid of low viscosity. Their selection is based on their performance, which is communicated by the pump manufacturer in the form of datasheets.

In some engineering processes, the working fluids are a mixture of two immiscible liquids which form an emulsion. A typical example is the pumps used in the oil industry, where water is produced together with oil, causing the formation of crude oil–water emulsions. In such cases, the complex rheological behavior of liquid–liquid two-phase flow and emulsion would significantly affect the flow pattern in the centrifugal pump and thus its performance. For instance, several studies have shown that the viscosity of the liquid–liquid dispersion and emulsion is higher than that of a single-phase fluid [1,2]. This viscosity depends essentially on the morphology of the emulsion and several parameters, such as the volume fraction of the coexisting phases, the size of the droplets of the dispersed phase, the shear stress, temperature, and the presence or not of a surfactant [3,4,5,6]. The size of the droplets, as well as the presence of a surfactant, affect the stability of the emulsion, where it has been shown experimentally that the more stable the emulsion, the greater its viscosity [7,8,9]. Pointing out that high shear stress leads to droplet fragmentation and small droplet diameters, resulting in a stable emulsion [10,11]. As pointed out in [12,13,14,15], the increase in pump speed and flow rate, which increases the shear rate, promotes the fragmentation of particles due to high turbulence levels. On the other hand, the process of inter-conversion of the phase configuration (phase inversion phenomenon) causes a dramatic rise in the emulsion viscosity, where it has been reported in the literature that the viscosity of the emulsion during the inversion process can be several times the viscosity of the single-phase oil constituting the emulsion [16,17,18]. This complex rheological behavior that is accompanied by viscosity fluctuations affects the flow pattern in the centrifugal pump and thus its performance. In this regard, determining the performance of centrifugal pumps carrying emulsion becomes imperative. Among the vast expanse of experimental investigations conducted on the effect of emulsion flow on pump performance [19], studies on how to determine the performance of these pumps carrying emulsion are one of the most sought-after areas of interest. In this regard, some researchers have used theoretical analysis or applied CFD as a numerical simulation tool to estimate pump performance under emulsion flow conditions. The analytical method can provide accurate predictions of the effects of liquid viscosity on pump performance when the pump geometry is known [20,21,22]. This method generally determines the energy performance by calculating the different losses based on a one-dimensional analysis, which has proven to be an efficient approach for pump design [23]. On this alignment, Zhu et al. [24,25] and Banjar et al. [26] proposed a semi-empirical correlation to predict the emulsion viscosity within the centrifugal pump, coupled with a mechanistic model for pump performance based on Euler’s equations and including all possible losses. The correlation gave a good estimate of the rheology of emulsions consisting of medium oil viscosities, while a divergence was noted for emulsions consisting of low oil viscosities. For the performance model, although it provided an accurate prediction with the experimental results at high rotational speeds, improvements were suggested at low speeds.

Due to the complexity involved in emulsion modeling, only a few attempts have been made to study the performance of a centrifugal pump under emulsion flow numerically. Becerra et al. [27] simulated the oil–water flow through a four-stage ESP using the volume of fluid (VOF) model and evaluated the inversion phenomenon during operating conditions. It was observed that higher oil volume fraction caused higher performance degradation. The CFD model adequately represented the behavior of the pump even though it does not consider the interaction between the phases. However, it does not capture the emulsion phase inversion. Valdes et al. [28] studied the behavior of a four-stage ESP carrying oil–water emulsion and its performance using different Eulerian approaches numerically. Namely, VOF, pure Eulerian, and a coupled CFD-PBM approach. The authors concluded that the VOF model did not give a good prediction of pump performance when the pumped emulsion has a colloidal morphology particularly far from the best efficiency point (BEP), however, the pure Eulerian model provided better predictions with differences in head and efficiency less than 7% and 10%, respectively. The CFD model, based on the population balance equation, provided acceptable results for the diluted emulsion (dispersed phase fractions up to 20%) but provided a significant difference in pump performance for the concentrated oil–water emulsion.

In general, the work in the literature on this topic has mainly focused on experimental investigations, while few analytical or numerical analyses have been performed. Moreover, these numerical studies have focused on the analysis of the overall performance of a multistage pump transporting emulsions, but no studies have focused on a volute centrifugal pump and the rheological behavior of these fluids. Hence, this study aims at analyzing the performance and flow characteristics of a volute centrifugal pump numerically when handling emulsions and oil–water mixtures at different water cut (WC) by considering their non-Newtonian rheological behavior, as well as to compare its performance when operating with Newtonian viscous oil. The rheological properties of the mixtures studied were measured experimentally under a shear rate ranging from 1 s^−1^ to 3000 s^−1^ and were fitted to conventional Cross and Carreau effective viscosity models. Therefore, the mixtures have been modeled and simulated as a single-phase fluid following non-Newtonian rheology. The numerical simulations were performed using a RANS approach for turbulence modeling. This work is an extension of the previous work [29]. The scope of the previous work was an analytical and CFD analysis of the performance of a centrifugal pump handling a concentrated oil-in-water emulsion (40O60W). This previous work was extended in this study by considering several types of emulsions. In addition, a more thorough internal flow analysis was performed here, relating the hydrodynamic performance of the pump to the rheological properties of the emulsion. The numerical results obtained showed that the viscosity plateau of the pseudoplastic behavior and the flow topology have a high impact on the pump performance. The smaller the value of ν_0_, the less frictional losses the fluid will experience, and conversely, the larger the ν_∞_, the fewer recirculation losses the fluid will experience. The recirculation and friction losses are related to the values of the upper and lower limits of the model, as well as the shear sensitivity reflected by the thinning behavior.

## 2. Numerical Modeling

The CFD simulations were performed with the open-source library OpenFOAM v1906 which uses a finite volume method (FVM) to discretize the fluid equations.

### 2.1. Geometry and Mesh

**Figure 1:**
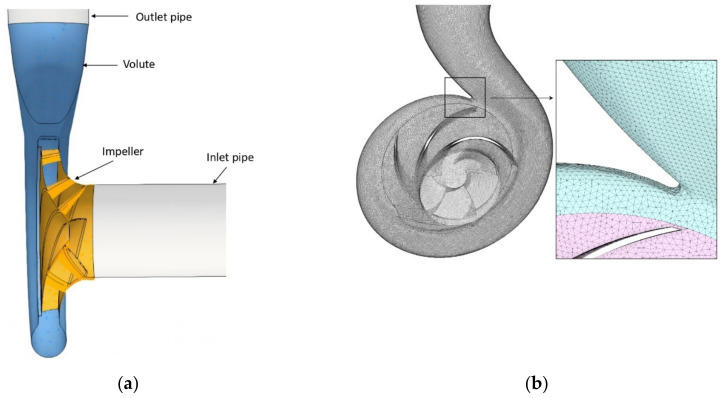
(**a**) Pump 3-D model and (**b**) fluid volume mesh.

The pump studied consisted of a five-bladed backward curved impeller and a volute. Its geometry considered four parts: the inlet pipe, the impeller, the volute, and the outlet pipe (Figure 1a). The nominal operating conditions of the pump were a rotational speed of 1470 rpm and a flow rate of 590 m^3^/h. The main geometric dimensions of the impeller, volute, and pump specifications are summarized in Table 1.

For the mesh generation, an unstructured mesh was generated due to the complex geometry of the centrifugal pump. Two types of mesh elements were considered: polyhedrons and prisms consisting of approximately 4 million cells in total. The hexahedral elements were employed in the inlet and outlet suctions, while the polyhedrons elements were employed in the impeller and volute. To capture the flow details near the flow domain boundaries, a structured mesh was used for the boundary layer of the rotating impeller. This led to an average y + < 5 and direct resolution of the viscous sublayer of the inner region.

**Figure 2:**
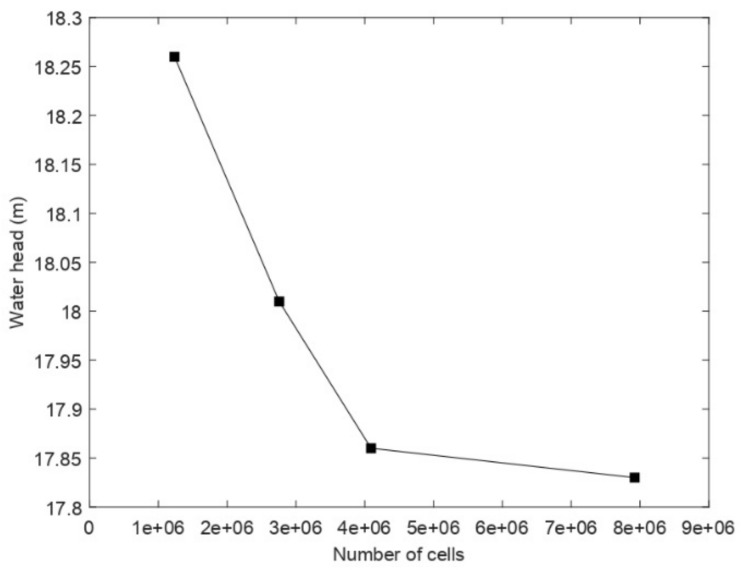
Grid sensitivity study.

To determine the most favorable number of cells to use for the simulations, a mesh independence test was performed. The centrifugal pump head considering water was taken as a reference parameter to determine the influence of the mesh size on the convergence to the exact solution (Table 2). Four different mesh sizes were evaluated (M1: coarse, M2: basic, M3: fine, and M4: ultrafine). The convergence criterion chosen was a relative head difference with a maximum value of 1%. Figure 2 shows the convergence of the calculated discharge pressure to an asymptotic value as the number of grids increases. The M3-grid (4 × 10^6^ grids) was considered sufficiently reliable to ensure non-mesh dependence and was used for the rest of the study. Figure 1b gives a general view of the generated mesh.

The quality of the mesh used to solve a problem is essential to the quality of the solution. Some essential parameters for the quality of the mesh were evaluated, including Skewness, Orthogonal Quality, and Aspect Ratio, with their values given in Table 3 for the mesh considered in this study.

### 2.2. Physical Model Specification

In this study, the flow field within the centrifugal pump was assumed to be incompressible, single-phase, isothermal, viscous, and turbulent. The mass and momentum equations (Equations (1) and (2)) are solved using the SIMPLE algorithm.

(1) $$\frac{\partial }{\partial t}\left(\rho \right)\;+\;\nabla \cdot \;(\rho \bar{u})\;=0$$

(2) $$\frac{\partial }{\partial t}\left(\rho \bar{u}\right)\;+\;\nabla \cdot \;\left(\rho \bar{u}⊗\bar{u}\right)\;=g\;+\;\nabla \cdot \bar{\left(\tau \right)}-\nabla \cdot \;\left(\rho R\right)$$

$\bar{\tau }$ is the averaged stress tensor and R is the Reynolds stress tensor. The Reynolds stress terms in RANS equations are modeled by the two transport-equation linear eddy-viscosity turbulence closure model k-Ɛ. The solved turbulent kinetic energy equation k and dissipation rate equation Ɛ are given by Equations (3) and (4), respectively. The motion of the rotational domain was modeled using the multiple reference frame (MRF) technique, in which the impeller is in the rotating frame, and the volute and the rest of the pump geometry in the stationary frame. This technique is a steady-state approximation where the effects of rotational motion are reproduced via source terms in the fluid equations. The information between the different regions is transferred by an arbitrary mesh interface (AMI), where the flow variables are assumed steady. This allows the flow field within the centrifugal pump to be predicted by steady-state calculations and saves simulation time.

(3) $$\frac{D}{Dt}\left(\rho k\right)\;=\;\nabla \cdot \;\left(\rho D_{k}\nabla k\right)\;+\;P-\rho \epsilon$$

(4) $$\frac{D}{Dt}\left(\rho \epsilon \right)\;=\;\nabla \cdot \;\left(\rho D_{\epsilon }\nabla \epsilon \right)\;+\;\frac{C_{1}\epsilon }{k}\left(P+C_{3}\frac{2}{3}k\nabla \cdot u\right)\;-\;C_{2}\rho \frac{\epsilon^{2}}{k}$$

(5) $$\nu_{t}=C_{\mu }\frac{k^{2}}{\epsilon }$$

The inlet and outlet boundary conditions were set to a fixed volumetric flow rate and static pressure of 0 Pa, respectively, and a no-slip velocity condition was imposed on all pump walls. By changing the volumetric flow rate, the performance curves of the centrifugal pump were acquired.

### 2.3. Numerical Model Validation

In the interest of assessing the accuracy of the CFD model, a standard case with water only was first considered. The numerical simulations were performed at a rotational speed of 900 rpm, as the experiment was realized under these conditions. The head developed by the pump was estimated as the difference between the surface averaged pressure at the outlet and the inlet. The head and efficiency were calculated following Equations (6) and (7), respectively.

(6) $$H=\frac{\Delta P}{\rho g}+\frac{v_{2}^{2}-v_{1}^{2}}{2g}+\Delta z$$

(7) $$\eta =\frac{\rho gQH}{\tau \;N}$$

**Figure 3:**
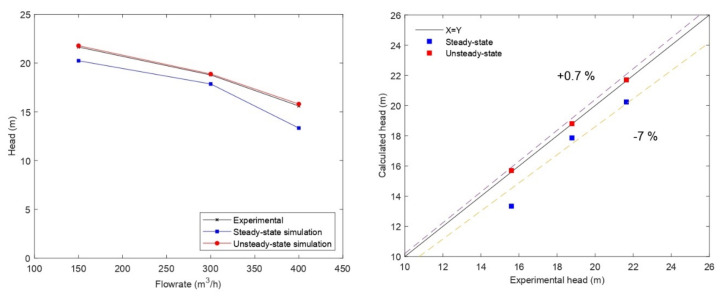
Comparison of pump head predicted by the CFD model with experimental data for water.

Figure 3 shows the comparison between the simulated pump head and the experimental data as a function of flow rate. Although the trend was well reproduced by the steady-state numerical model, the model underestimated the head values compared to the experimental data. Nearly all data points were within a 10% discrepancy. The differences are related to the assumption of a steady-state simulation to model the flow field inside the centrifugal pump. Therefore, only one impeller position was considered and the interaction between the impeller blades and the volute tongue was not considered. In a previous study, Asuaje et al. [30] verified the effects of the interaction between the impeller and the volute for the same pump, by performing steady-state computations of several impeller positions. The author found that this position influences the head obtained and that the amplitudes of the pressure fluctuations reached 27% of the average pressure generated by the impeller.

To further investigate this point and to account for the different positions of the impeller, a simulation of the flow field in the pump in an unsteady regime was considered with the same mesh. The PIMPLE algorithm (merged PISO-SIMPLE) was used to solve the continuity and momentum equation. The stator–rotor interaction was modeled using a sliding grid approach. This transient method rotates the rotor part of the mesh relative to the stator part at each time step and the local flows were transferred using the AMI interface. Thus, the interaction between the impeller and the volute was fully resolved. The results of the unsteady simulation of the water flow field within the pump are shown in Figure 3. The comparison with the experimental data showed that the unsteady numerical results overestimated the head by about 0.7%. The numerical model did not consider the effect of the tip clearances of the pump, which accounts for a maximum of 2.5% of the head losses. In the light of these results, the current numerical model can be considered satisfactory in terms of accuracy, thus validating the numerical model.

For the sake of computational resources, simulations of the flow fields inside the studied centrifugal pump handling emulsions of different concentrations were performed at steady-state conditions. The objective was to investigate the internal flow within the pump handling emulsions, modeled as non-Newtonian fluids, along with the performance degradation of the pump carrying this type of fluid. The influence of unsteady phenomena was not included in this study.

### 2.4. Emulsion Rheology

The flow systems considered in this study corresponded to the emulsions studied experimentally by Valdes et al. [31]. The authors studied two-phase mixtures of sunflower oil and water at 9 different phase compositions, ranging from 90%–10% O/W to 10%–90% O/W, with 10% increments. The rheological properties of these mixtures were measured experimentally under a shear rate ranging from 1 s^−1^ to 3000 s^−1^, which covers the range of the shear rate of the studied pump. Additional information on the experimental procedure can be found in [31]. The results of the rheological characterization were fitted to conventional Cross and Carreau effective viscosity models given by Equations (8) and (9). Therefore, this study modeled the two-phase emulsions as a single-phase fluid with non-Newtonian properties.

(8) $$\frac{\mu_{eff}-\mu_{\infty }}{\mu_{0}-\mu_{\infty }}={\left[1+{\left(K_{C}\dot{\gamma }\right)}^{n_{C}}\right]}^{-1}$$

(9) $$\frac{\mu_{eff}-\mu_{\infty }}{\mu_{0}-\mu_{\infty }}={\left[1+{\left(\lambda_{t}\dot{\gamma }\right)}^{2}\right]}^{\frac{n_{car}-1}{2}}$$

**Figure 4:**
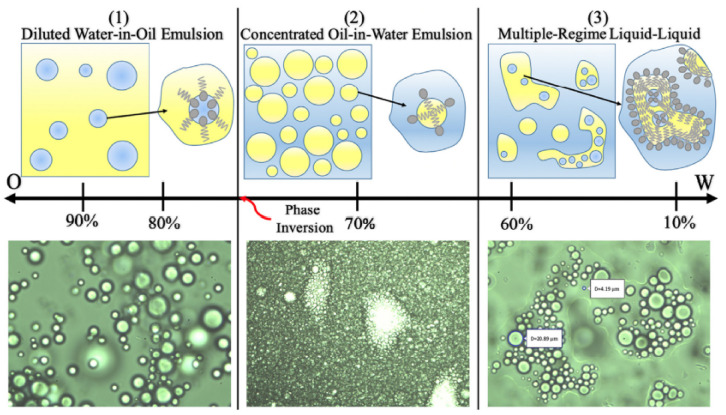
Oil–water phase morphology and distribution map for the studied mixtures from Valdes et al. [31]. Phase composition (%*v/v*) given with respect to the oil phase. The top row gives a schematic representation, while the bottom row provides representative optical microscopic images of each region.

$K_{C}$ and $n_{C}$ are the Cross time and Cross rate constant, respectively. $\lambda_{t}$ and $n_{car}$ are the relaxation time and the power index, respectively. $\mu_{0}$ and $\mu_{\infty }\;$ are the viscosity for zero shear rate and very high shear rate. The studied emulsions and its properties are presented in Table 4. Three-phase morphologies were identified (Figure 4): (1) the dilute, pseudo-stable W/O emulsion at high oil fractions (>80%), exhibiting quasi-Newtonian behavior with a slight tendency to shear thinning at very high shear rates. (2) a concentrated pseudo-stable O/W emulsion where phase inversion occurred at 70% oil volume, characterized by higher viscosity and tendency to shear thinning. (3) Multi-regime emulsion at high water fractions (>40% water), with a slight tendency to shear thinning (the reader is referred to the article [31] for a better understanding of the fluid characterization performed and the interpretation of the results).

## 3. Results

### 3.1. Centrifugal Pump Performance

**Figure 5:**
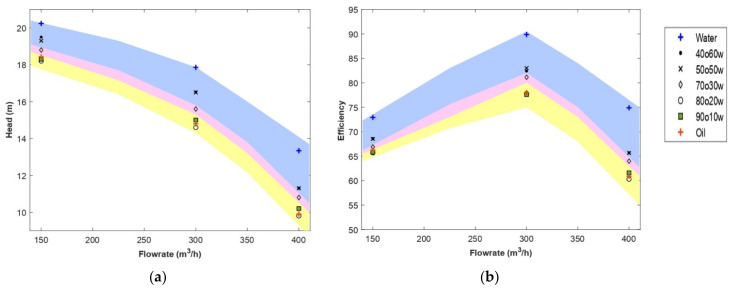
CFD head (**a**) and efficiency (**b**) curves of the studied emulsions. Yellow, pink, and blue shaded areas correspond qualitatively to regions 1, 2, and 3, respectively, as classified in Figure 4.

Figure 5 shows the head developed by the pump and the performance obtained for the selected compositions in each region. Simulations were performed on a flow rate ranging from 100 m^3^/h to 400 m^3^/h, with a step of 50 m^3^/h for two emulsions. Since the degradation varied only slightly with the flow rates, in this study, only the nominal flow rate, the lower and upper limits of flow range were considered. Thus, the analysis considered the entire operating range of the centrifugal pump. The regions shown previously in Figure 4 are highlighted to relate the composition and rheological properties of the emulsion to the pump operation. First, a clear transition was observed in the head and performance curves before and after the phase inversion point (region 2). The pump performance developed for indirect emulsions (region 1) and oil was significantly close. However, the head obtained for the oil was higher than the one obtained for the 80O20W emulsion. The head difference between the two fluids decreased as the flow rate increased to approximately the same head value at high flow rates. On average, the 80O20W emulsion obtained a head degradation of 10% in underflow, which increased to 26% in overflow. On the other hand, the oil led to a head degradation of 9% in underflow, which increased to 25% in the overflow. This difference in performance is related to the composition of the emulsion and its viscosity. The 80O20W emulsion was close to the phase inversion point, where high viscosity is observed compared to other emulsions outside the phase inversion zone so that losses increase, and pump performance deteriorates. On the other hand, the 80O20W emulsion followed a shear-thinning behavior, so its viscosity decreased as the shear rate increased to reach a lower viscosity limit which was close to the oil viscosity (Table 4). This explains the decrease in the head difference at high flow rates.

Comparing the performance of the pump when handling 90O10W emulsion with the performance of the pump when handling oil, it was observed that at low flow rates, the head developed for oil was higher than that developed for emulsion. At nominal flow and overflow, the opposite occurred. Given the shear-thinning behavior of the emulsion, its viscosity will tend to decrease at high shear rates. This implies that the effective viscosity of this emulsion will be much lower than the viscosity of the oil ($\nu_{oil}$ > $\nu_{\infty \;\left(90O10W\right)}$), thus decreasing the hydraulic losses encountered in the pump and resulting in higher head values.

**Figure 6:**
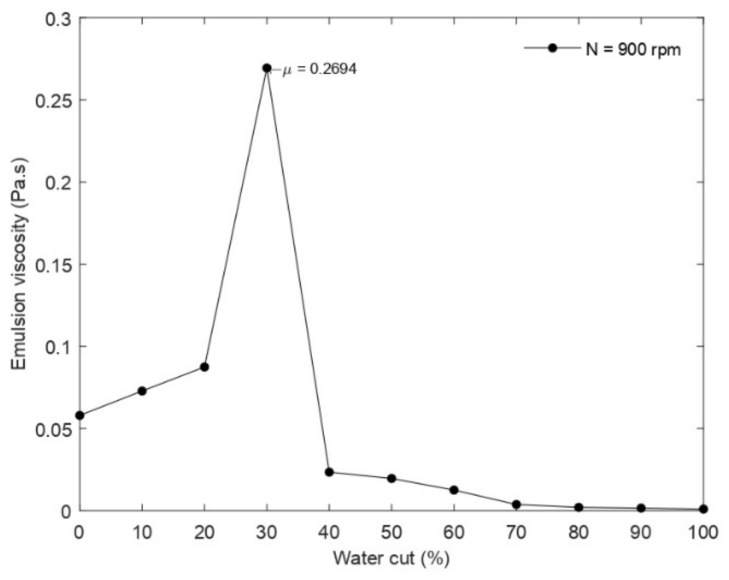
Viscosity curve vs. water cut and inversion point (graph made from the experimental data of Valdes et al. [31] by selecting the shear rate corresponding to the studied pump).

As for region 2, a viscosity peak of the emulsion was observed at phase inversion as shown in Figure 6, but the performance of the pump carrying this emulsion was better than that of the pump handling region 1 emulsions and oil (Figure 5). This was due to the strong shear-thinning behavior of the emulsion and the lower limit of its effective viscosity range. Emulsion 70O30W will undergo a significant viscosity drop until viscosity values lower than those of the region 1 emulsions are reached at the same shear rates. As a result, lower losses will be generated, and pump performance will be improved.

The pump performance degradation observed when pumping the emulsions in region 3 was noticeably close, especially at the nominal flow rate. This was due to the close viscosity range of the two emulsions and their sensitivity to shear rate governed by the coefficients k and n (Table 4). Emulsion 50O50W has a higher viscosity at low shear rates than emulsion 40O60W, and at high shear rates, their viscosities were almost equal. In addition, emulsion 40O60W had a more pronounced shear-thinning behavior (k > k, n ≈ n), so that over the entire shear rate range, the viscosity of emulsion 40O60W will be lower than that of emulsion 50O50W. This explains the slightly better performance obtained for emulsion 40O60W at underflow and nominal flow rates. Nevertheless, both emulsions will see their effective viscosities decrease sufficiently to reach the lower bounds ($\nu_{\infty \;}$) at very high shear rates. Therefore, both emulsions developed the same performance at overflow.

**Figure 7:**
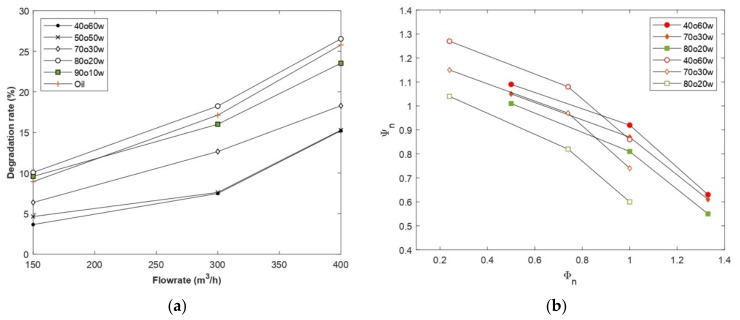
(**a**) Degradation rate of the pump head of the studied compositions and (**b**) Curves of normalized head coefficient as a function of the normalized flow coefficient for CFD results and Valdes [31] experimental data.

Another interesting point to discuss is the rate of head decline. A larger drop in the head curve at higher flow rates was observed in region 1 (degradation rates between 24% and 26%, corresponding to the 90O10W and 80O20W emulsions, respectively) when the oil fraction was large (>70%) (see Figure 7). This suggests that systems with higher oil fractions generate higher frictional losses, which is consistent with the increase in the effective viscosity range shown in Table 4 and especially the lower bound of the viscosity range. The centrifugal pump generated high shear rates so that the fluids reached the lower Newtonian plateau. Thus, the increase in frictional losses is directly related to the lower bound of the viscosity range of each emulsion. This explains the minimal change in head obtained by increasing the oil fraction by 10% in region 3 ( ν∞ 40O60W ν∞ 50O50W ≈1), compared to the sharp drop observed by increasing the oil fraction by 10% in region 1 ( ν∞ 80O20W ν∞ 90O10W ≈2.8). This also explains the more pronounced degradation in performance of the 80O20W emulsion compared to oil ($\nu_{oil}\;$ < $\nu_{\infty \;\left(80O20W\right)}$), as shown in Figure 7. A similar analysis could be made from the pump efficiency results, where the pump efficiency decreased as the volume fraction of the oil phase increased, which can be explained by the increase in the effective viscosity range of the different emulsions.

A comparison between the CFD results and the experimental data from the reference paper is presented in Figure 7b. This figure shows the normalized head versus normalized flow rate for the CFD results and Valdes’s experimental data based on a multistage pump with a specific speed twenty times the specific speed of the studied pump. As can be noticed, the graph trend is similar for both results. Another interesting observation is the rate of head deterioration rate. The head curve for emulsion shown in Figure 7b showed a more pronounced drop at higher flow rates. This observation is in accordance with the experimental results of Valdes et al. [31], where, for example, a 4% performance degradation was noted before BEP for 40O60W emulsion, increasing up to 14% at this point. In this study, the degradation rate was 9% before BEP and increased up to 17% after this point. First, this difference in deterioration was mainly due to the difference in pump geometry and operating conditions that affect the hydraulic losses in terms of quantity. Second, despite the shear-thinning behavior of the emulsion, its high effective viscosity led to degradation of pump performance, primarily through increased frictional losses at high flow rates.

### 3.2. Internal Flow Analysis

**Figure 8:**
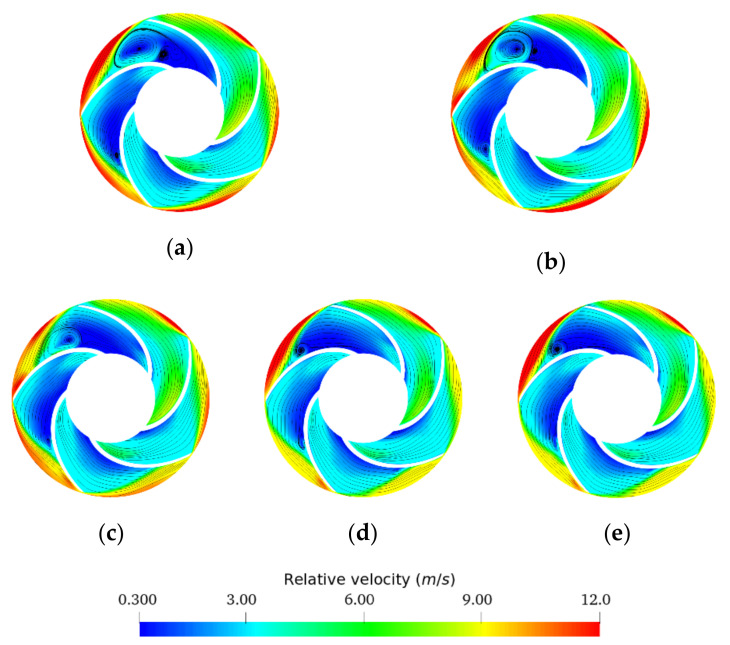
Relative velocity profiles and streamlines on the impeller at 0.5 BEP for oil fraction (%*v/v*): (**a**) 0%, (**b**) 50%, (**c**) 70%, (**d**) 80%, (**e**) 100%.

**Figure 9:**
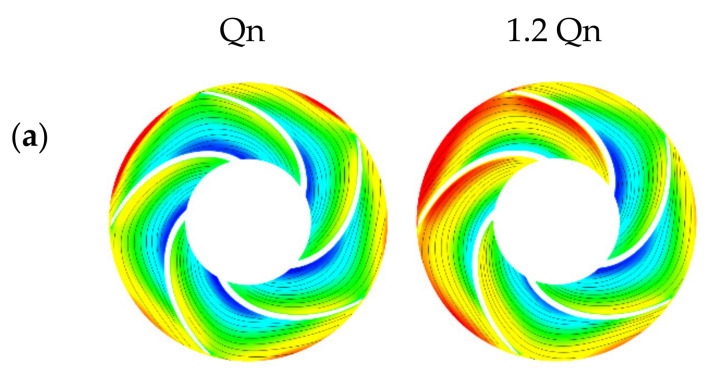

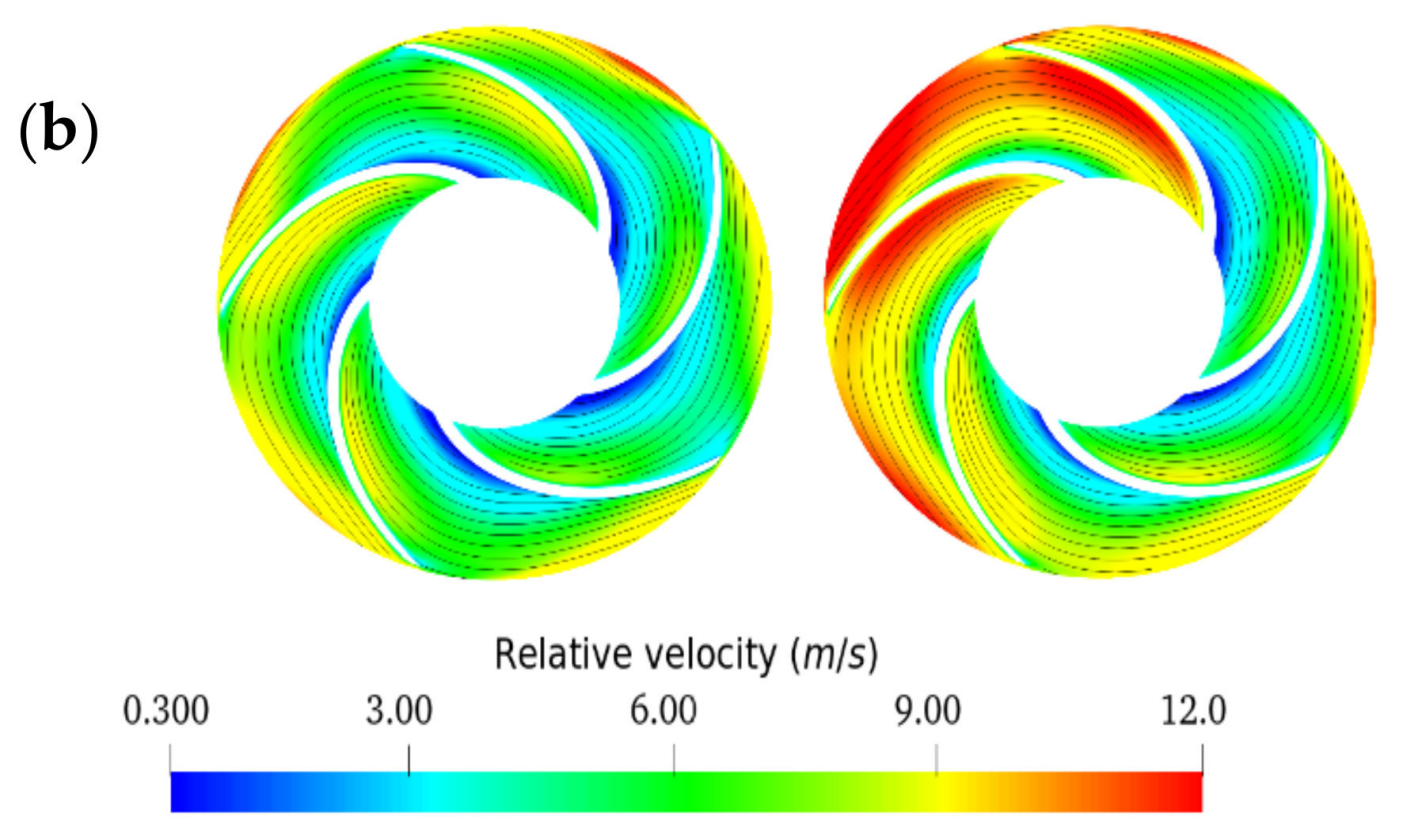
Relative velocity profiles and streamlines on the impeller at BEP and 1.2 BEP for (**a**) region 1 emulsion and (**b**) region 3 emulsion (Since the flow structure was almost identical for all fluids from the nominal flow rate, the streamlines and velocity vectors are represented only for two emulsions, one from region 1 and another from region 3).

In this section, a correlation between the rheological behavior of the studied emulsions with the internal flow characteristics within the centrifugal pump will be established. For this purpose, the relative velocity profiles, streamlines, and velocity vectors in the impeller as well as the absolute velocity profiles in the volute will be analyzed for each emulsion and compared to the velocity profiles of water and oil (Figure 8 and Figure 9).

Emulsions from the same region show very similar behavior, so the streamlines and velocity vectors of a single emulsion from each region were selected to show the flow through the pump.

First, all recirculation zones appeared at underflow for all fluids and the inter-blade spaces in contact with the volute nozzle (Figure 8), highlighting the asymmetric operation of the pump. As the impeller periodically swept the volute nozzle, the fluid discharged by the impeller interacts with the volute nozzle, producing a large amount of energy dissipation, resulting primarily from impact and recirculation losses.

From Figure 8, significant recirculation zones in the impeller for the emulsions in region 3 were observed. This was due to the low viscosity range of these two emulsions, where the high shear rate generated by the impeller drops the viscosity of these fluids to the lower Newtonian plateau, thus exhibiting a similar behavior to water.

For the 70O30W emulsion (Figure 8c), smaller areas of recirculation than those generated by the region 3 emulsions (Figure 8b) appeared but larger than those of the region 1 emulsions (Figure 8d). This was due to the strong shear-thinning behavior of the emulsion (high k, high n) despite the very high viscosity range at low shear rates. As mentioned earlier, the studied centrifugal pump generates high shear rates that lead to a sharp decrease in the effective viscosity of this emulsion until it reaches the lower Newtonian plateau. Further, if we compared the lower limit of the viscosity ranges of the different emulsions (Table 4), we observed that the viscosity of the emulsion 70O30W at high shear rates was between the values of the viscosities of the emulsions in regions 1 and 3.

Region 1 emulsions had a higher effective viscosity range caused by the higher oil phase concentrations, thus influencing the velocity field and vortex formation. For emulsions in this region, a behavior similar to that of oil was observed.

This indicates that recirculation loss with this type of centrifugal pump decreases with increasing fluid viscosity and that the most influential factors on recirculation loss when dealing with non-Newtonian fluids is the lower limit of the effective viscosity range, as well as the strong tendency to shear thinning of the fluid to reach this limit. This is especially true when comparing the flow field of oil and water, where smaller recirculation zones are observed for oil. Similar conclusions could be drawn when looking at the magnitude of the relative velocities (especially at the blade passage inlet).

From the results obtained at underflow, it was noted that the relative velocities increased as the volume fraction of the oil phase increased, this at the suction blade passages and the impeller inlet. This result indicates that incidence losses increase with viscosity, participating in the head degradation observed in Figure 5. Another observation concerns the direction of the velocity vectors at the impeller exit. For the emulsions of region 3 and water, we noticed a deviation of the vectors from the direction of the blade profile, for all the blades of the impeller. For emulsions with a high oil volume fraction (>70%) and pure oil, there was an accentuated deviation of the velocity vectors from the direction of the blade profile in contact with the tip of the volute and much less accentuated in the other blades of the impeller. This was caused by the high viscosity of the oil and emulsions in region 1, and the non-symmetrical geometry of the pump (presence of the volute).

For all fluids, as the flow rate increased, the relative velocity values increased, and the streamlines tended to better follow the blade profile. One can note that the relative velocities at the impeller inlet were almost identical for all fluids and the velocity distribution in the impeller was almost similar (Figure 9). As a result, recirculation and impact losses became significantly lower and friction losses became more influential as the flow rate increased for all fluids.

**Figure 10:**
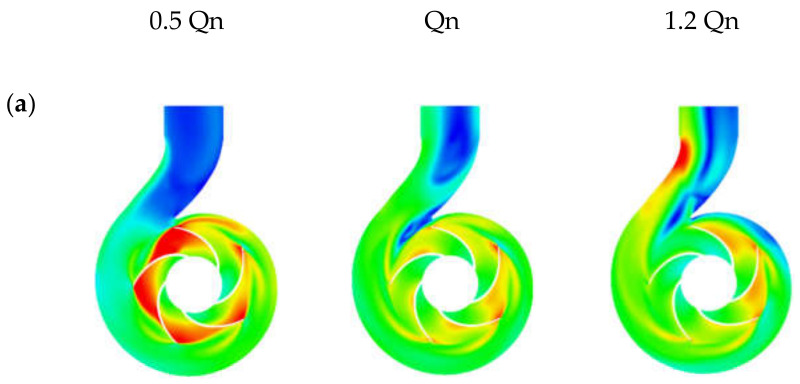

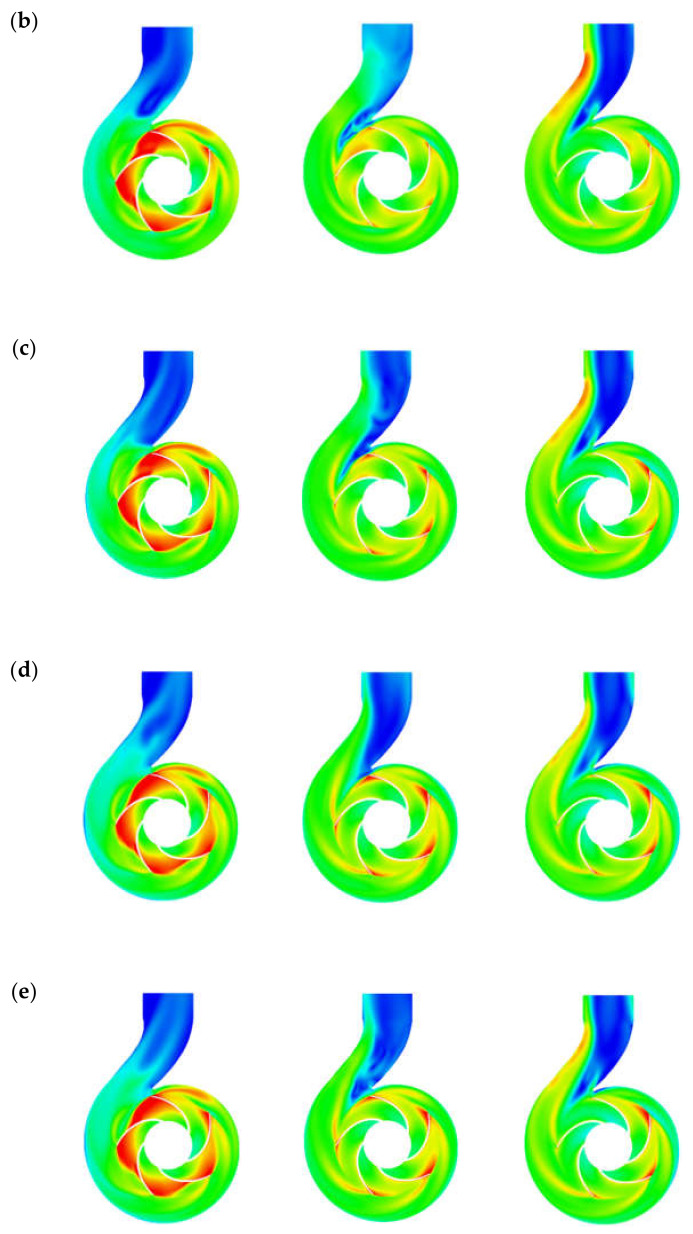

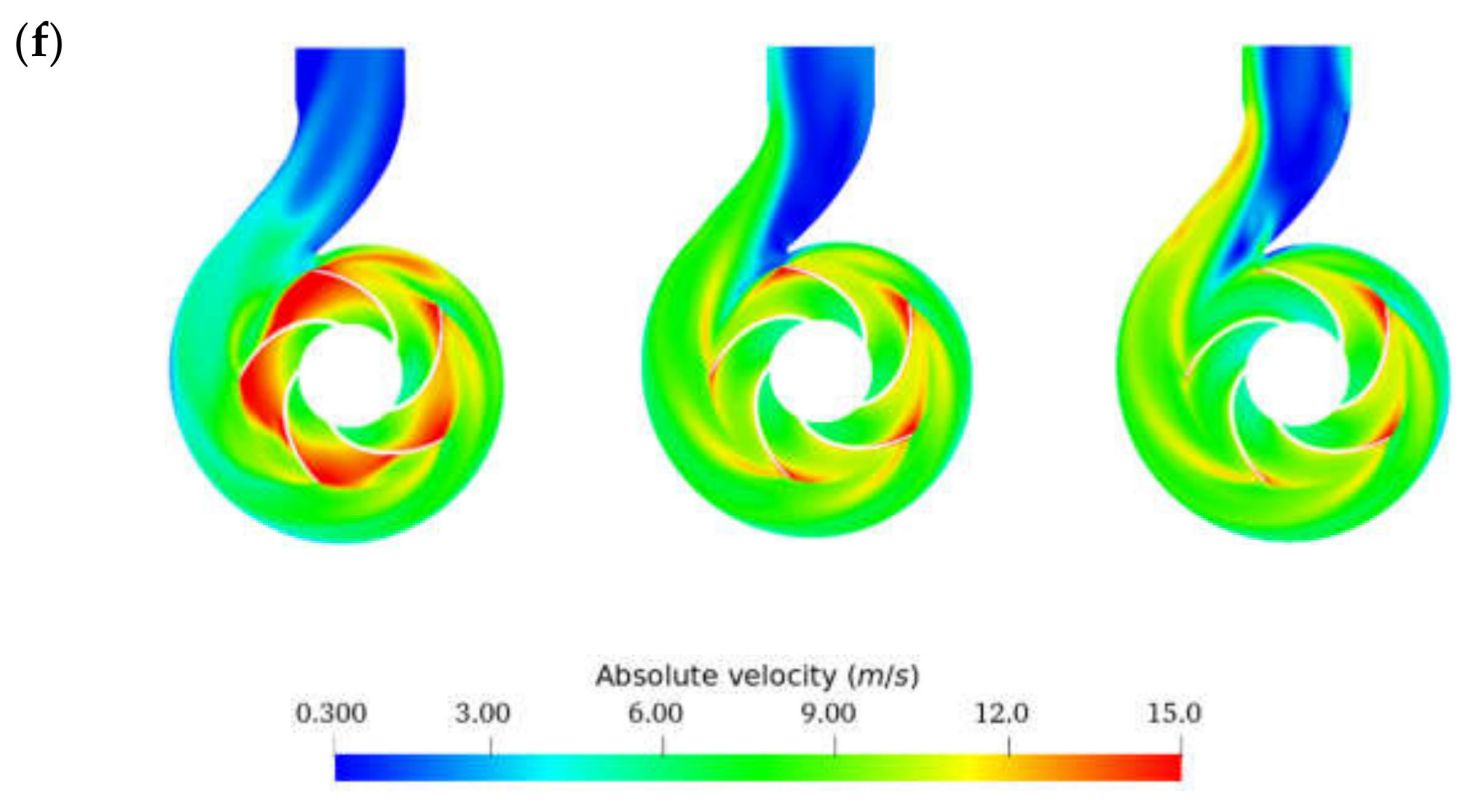
Absolute velocity profiles and streamlines on the impeller at 0.5Qn, Qn, and 1.2Qn for oil fractions (%*v/v*): (**a**) 0%, (**b**) 50%, (**c**) 70%, (**d**) 80%, (**e**) 90%, and (**f**) 100%.

Concerning the flow profile in the volute, at the nominal flow rate, the oil, and the region 1 emulsions were correctly channeled (Figure 10 (d–e)). The water and the other emulsions showed swirls at the volute neck and its outlet (Figure 10 (a–c)). These vortex areas were larger when water was considered. We observed that when the fraction of oil increased, the two vortex zones increased and coalesced. These figures show a transition from chaotic to uniform flow at the volute neck as the fluid viscosity increased (see fluid viscosity range limits in Table 4). This was especially true when comparing the flow profile of emulsion 90O10W with that of emulsion 80O20W. A chaotic flow in the neck of the volute was observed for emulsion 90O10W, while emulsion 80O20W exhibited a uniform flow given its high effective viscosity range. The same observation could be made for the underflow. However, at the overflow, the flow profiles in the volute were almost similar for all fluids. Noting a zone of acceleration at the neck of the volute (Figure 10 (1.2Qn)), which was dampened as the viscosity of the fluid increased. Therefore, the flow in the vicinity of the volute nozzle was strongly influenced by the viscosity of the fluid and the variation of the flow. These vortices and dead zones contributed to hydraulic losses by causing the fluid to lose kinetic energy.

### 3.3. Effective Viscosity Variation and Shear Stress Profiles

**Figure 11:**
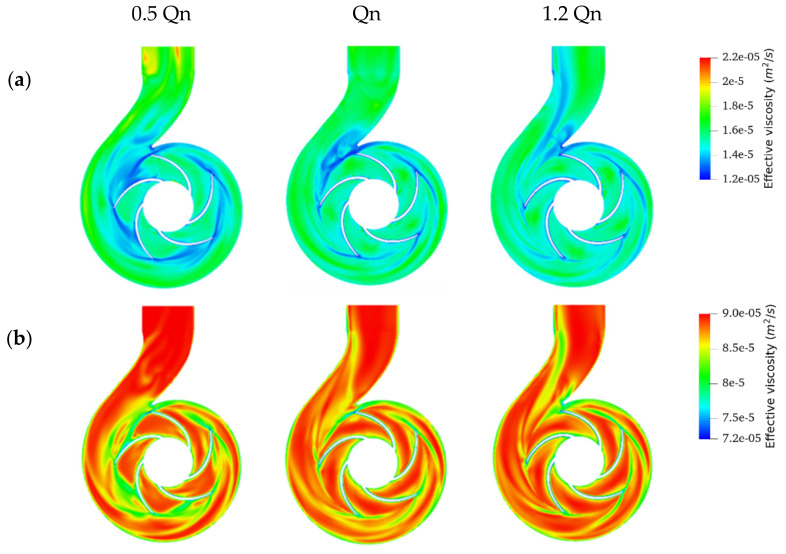
Effective viscosity profiles for oil fractions (%*v/v*): (**a**) 50% and (**b**) 80% operating at 0.5 BEP, BEP, and 1.2 BEP.

**Figure 12:**
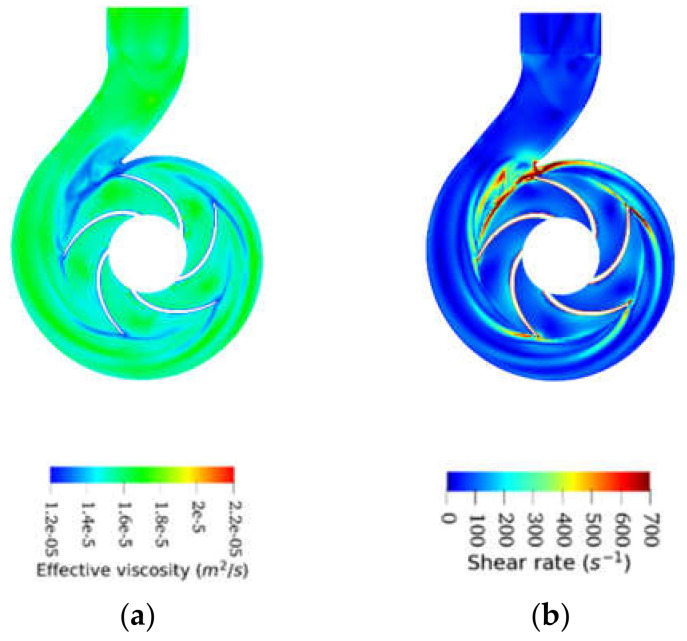
Effective viscosity profile (**a**) compared with shear rate profile (**b**) for 40O60W emulsion operating at BEP.

As expected, the effective viscosity for all emulsions dropped as the operating flow rates increased (Figure 11), in accordance with their shear-thinning behavior. This shear-thinning behavior is highlighted in Figure 12, which shows the effective viscosity profile compared with the shear rate profile for a typical emulsion operating at BEP. As can be seen, the high shear stress regions in Figure 12b (volute neck, blade surfaces, and at the impeller-volute contact interface) corresponded to the low viscosity region in Figure 12a.

**Figure 13:**
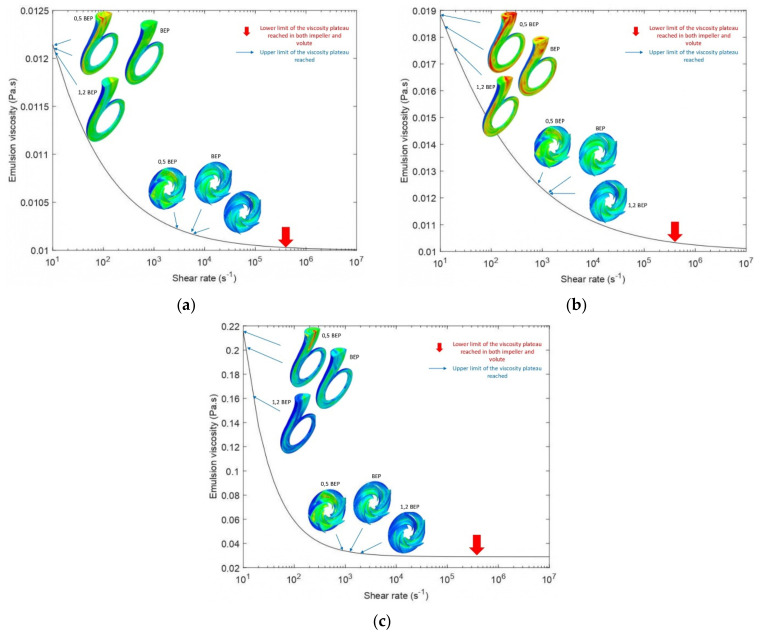

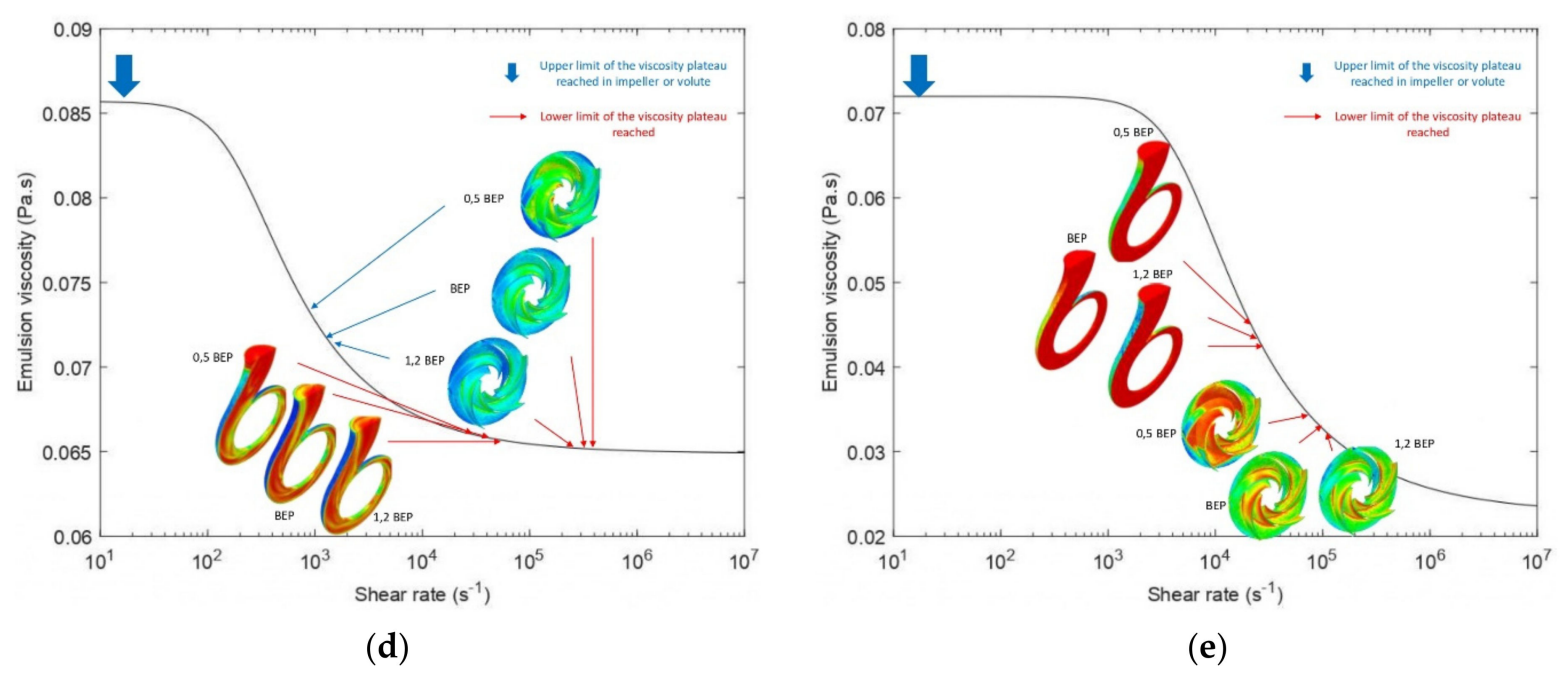
Viscosity range in the impeller and volute at operating conditions 0.5 BEP, BEP, and 1.2 BEP for oil fractions (%*v*/*v*): (**a**) 40%, (**b**) 50%, (**c**) 70%, (**d**) 80%, (**e**) 90%.

The second crucial result was that the effective viscosities observed at the outlet section and the volute were significantly higher than those observed at the rotating region for the region 1 emulsions fitted to the Cross model, for all flow rates considered, as shown in Figure 11a. In sum, higher viscosities were observed away from the impeller due to the lower shears exerted on the fluid in this region [29]. In this figure, it can be seen, for example, that the maximum viscosity of emulsion 50O50W at the impeller was about 1.24 × 10^−5^ m^2^/s^2^, while its upper limit in the volute reached 1.91 × 10^−5^ m^2^/s^2^. It was also observed for emulsions of this region, that the lower limit of the expected viscosity range at the impeller and volute was the same, and the flow rate did not affect this value. On the other hand, the upper limit of the viscosity range varied with the flow rate and differed between the rotating and static parts (Figure 13a,b).

The region 1 emulsions modeled by Carreau showed a different behavior, their viscosity dropped at the walls to very low values, but small viscosity variations were observed away from the walls throughout the pump Figure 11b. The lower limit of the viscosity range at the impeller and volute varied with the flow rate and differed between the rotating and static parts. However, the upper limit of the viscosity range was the same, and the flow rate did not affect this value (Figure 13d,e).

If we examined the influence of the flow rate on the viscosity profile in the rotating part and the static part for all emulsions, we observed that the rate of change in viscosity in the rotating part was lower than the rate of change in viscosity in the static part. This suggests that the shear rate in the rotating region is dominated by rotational speed and is less sensitive to flow variations. In contrast, in the other regions of the pump, the shear rate is very sensitive to flow variations.

Figure 13 shows the viscosity variations of the different emulsions in the pump at different flow rates. The effective viscosity range of emulsion 70O30W (region 2), bounded by $\nu_{\infty }$ at high shear rates, was significantly larger than that of emulsions of region 3, despite its greater tendency to shear thinning. This implies that emulsion 70O30W will exhibit greater viscosity decreases. The effective viscosities will yet still be higher than those of the two emulsions in region 3 but lower than those of emulsions in region 1. This explains the lower pumping performance obtained when handling emulsion 70O30W relatively to the other emulsion, as presented previously in Section 3.1.

On average, the effective viscosity of the 90O10W emulsion was 4.06 × 10^−5^ m^2^/s^2^ on the impeller wall and 5.47 × 10^−5^ m^2^/s^2^ on the volute wall. The average viscosities of this emulsion in walls of the rotating and stationary regions were lower than the viscosity values of the oil. This means that the oil will generate greater frictional losses than this emulsion. On the other hand, moving away from the walls, the effective viscosity slightly increased to 7.53 × 10^−5^ m^2^/s^2^ and 7.59 × 10^−5^ m^2^/s^2^ in the impeller and volute, respectively, which are values higher than the viscosity value of the oil. This led to smaller recirculation zones for the emulsion than for the oil. This explains the higher performance obtained by the pump when handling emulsion 90O10W compared to oil. In contrast, emulsion 80O20W showed average values of 6.82 × 10^−5^ m^2^/s^2^ at the impeller and 8.89 × 10^−5^ m^2^/s^2^ at the volute. These viscosity values are higher than the viscosity value of the oil, which explains the more pronounced performance degradation when handling this emulsion.

For the emulsions in region 3, the effective viscosity of the two emulsions was remarkably close at high flow rates, noting an average effective viscosity over the whole domain of 1.06 × 10^−5^ m^2^/s^2^ and 1.11 × 10^−5^ m^2^/s^2^ for the 40O60W and 50O50W emulsions, respectively. A slight difference in viscosity values at low flow rates was noted, given the upper limit of their viscosity range. This explains the very close pump performance observed previously when handling these two emulsions.

**Figure 14:**
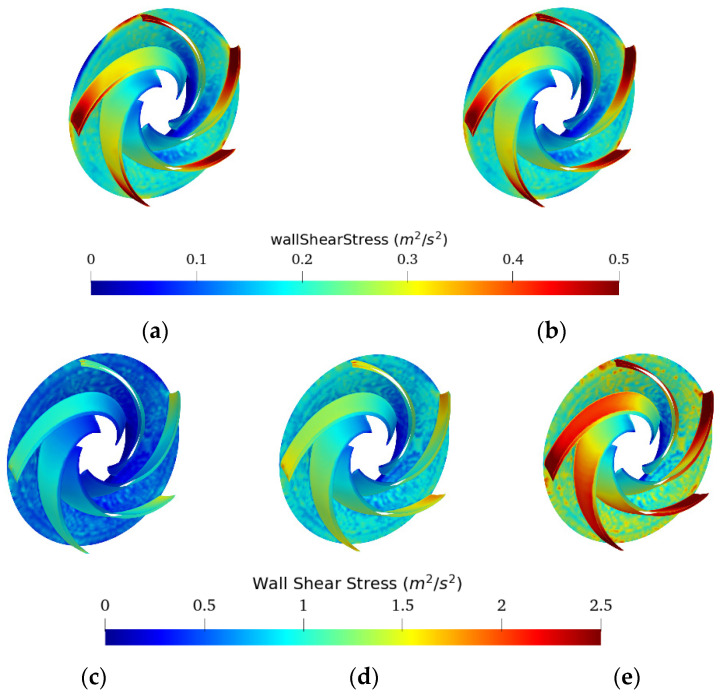
BEP Wallshear Stress (divided by density) profiles on the impeller for oil fractions (%*v*/*v*): (**a**) 0%, (**b**) 40%, (**c**) 70%, (**d**) 90%, (**e**) 100%.

From Figure 14, it can be seen that the shear stresses generated on the impeller were significantly higher for oil and emulsion 80O20W compared to other emulsions and water. The average shear stresses of emulsion 80O20W oscillated between 800 and 2800 Pa, that of oil oscillated between 700 and 2300 Pa, that of emulsion 70O30W oscillated between 476 and 1200 Pa, while the two emulsions of region 1 and water showed a maximum of 700–850 Pa. Furthermore, the shear-thinning behavior of all fluids was again highlighted in this figure; the shear stresses generated at the impeller for oil and emulsion 80O20W were almost the same (**ν**_(**∞** (**80O20W**)_ ⁄ **ν_oil_** ≈ 1), and so for the shear stresses generated for the two emulsions of region 3. This suggests that non-Newtonian fluids behave like viscous Newtonian fluids in the rotating part independently of their sensitivity to shear-thinning, with viscosities near the lower Newtonian plateau, given the high shear rates generated by rotational speed.

The centrifugal pump studied generates high shear rates in the rotating part so that the non-Newtonian fluid behaves like viscous Newtonian fluids whose viscosity is near the lower Newtonian plateau.

## 4. Conclusions

In this study, CFD analysis was performed to investigate the performance and flow characteristics of a centrifugal pump handling emulsions of oil–water mixtures at different water cuts. The mixtures were modeled and simulated as a single-phase fluid following shear-thinning non-Newtonian rheology. The conclusions of this study are summarized below:

1. The pump head curves illustrated a general progressive deterioration in performance as the oil fraction increased, except for the composition at the inversion zone. Despite the high effective viscosity of this composition, the strong tendency of the fluid to shear-thinning and the high shear rates of the pump caused the viscosity to decrease sharply. Pointing out that the head developed by the pump at a low volume fraction of the dispersed phase (up to 20% WC) is almost identical to that developed by the continuous phase.
2. The smaller the value of ν_0_, the less frictional losses the fluid will experience, and conversely, the larger the ν_∞_, the fewer recirculation losses the fluid will experience. As well as the recirculation and friction losses are related to the values of the upper and lower limits of the model; they are also related to the shear sensitivity reflected by the thinning behavior (value of k and n).
3. Non-Newtonian behavior of the emulsions was observed with a wide range of effective viscosity in the volute. In contrast, the emulsions exhibited minor non-Newtonian characteristics and/or a small effective viscosity range in the impeller due to the high shear rate generated by rotation in this region.

## Figures and Tables

**Table 1:** NS32 specifications.

	Parameter	Value
Impeller		
Inlet diameter (mm)	$D_{1}$	150
Outlet Diameter (mm)	$D_{2}$	408.4
Inlet blade width (mm)	$b_{1}$	85.9
Outlet blade width (mm)	$b_{2}$	42
Inlet blade angle (°)	$\beta_{1}$	70
Outlet blade angle (°)	$\beta_{2}$	63
Number of Blades	$n_{blades}$	5
Blade thickness (mm)	e	8
Volute		
Diameter (mm)	$D_{3}$	436
Base width of the volute	$b_{3}$	50
Pompe NS32		
Nominal Head (m)	$H_{opt}$	49
Rotational speed (rpm)	N	1470
Nominal Flowrate ( $m^{3}$/h)	$Q_{opt}$	590
Specific Speed	$N_{s}$	32

**Table 2:** Mesh size used.

Characteristics	Number of Cells
M1	1,230,000,
M2	2,750,000,
M3	4,091,000,
M4	7,900,000,

**Table 3:** Parameters evaluated for the quality of the mesh.

Parameter	Max Aspect Ratio	Max Non-Orthogonality	Max Skewness
Value	349	7.95	3.13

**Table 4:** Measured density, Cross/Carreau fitting parameters for each composition studied, data from [31].

Phase Composition (% *v/v* oil)	Viscosity Model	ρ (kg/m^3^)	$n_{C}/n_{car}\;$ (-)	$K_{c}\;(s^{n})/\lambda_{t}(s)$	$\nu_{0}\;(m^{2}/s)$	$\nu_{\infty }\;(m^{2}/s)$	ν (m^2^/s)
100	Newtonian	922.0	-	-	-	-	6.29 × 10^−5^
90	Carreau	947.8	0.471	2.03 × 10^−4^	7.59 × 10^−5^	2.40 × 10^−5^	-
80	Carreau	952.2	0.421	5.30 × 10^−3^	9.01 × 10^−5^	6.82 × 10^−5^	-
70	Carreau Cross	953.0	0.339 0.801	45.88 23.39	1.57 × 10^−2^	3.14 × 10^−5^	-
50	Cross	966.9	0.339	1.94	4.44 × 10^−5^	1.03 × 10^−5^	-
40	Cross	978.3	0.416	21.06	3.27 × 10^−5^	1.02 × 10^−5^	-
0	Newtonian	1000.0	-	-	-	-	1.00 × 10^−6^

## Data Availability

Data available in a publicly accessible repository that does not issue DOIs. Publicly available datasets were analyzed in this study. This data can be found here: [https://www.dropbox.com/sh/mdk6zn2ly77v0jt/AAB3NyGtoCBbE08v19aZP0jTa?dl=0], accessed on 3 December 2021.
